# Where Do We Start? Building Consensus on Drivers of Health Sector Corruption in Nigeria and Ways to Address It

**DOI:** 10.15171/ijhpm.2019.128

**Published:** 2019-12-15

**Authors:** Obinna Onwujekwe, Charles T. Orjiakor, Eleanor Hutchinson, Martin McKee, Prince Agwu, Chinyere Mbachu, Pamela Ogbozor, Uche Obi, Aloysius Odii, Hyacinth Ichoku, Dina Balabanova

**Affiliations:** ^1^Health Policy Research Group, College of Medicine, University of Nigeria Enugu Campus, Enugu, Nigeria.; ^2^Department of Health Administration and Management, College of Medicine, University of Nigeria Enugu Campus, Enugu, Nigeria.; ^3^Department of Psychology, University of Nigeria, Nsukka, Nigeria.; ^4^London School of Hygiene and Tropical Medicine, London, UK.; ^5^Department of Social Work, University of Nigeria, Nsukka, Nigeria.; ^6^Department of Community Medicine, College of Medicine, University of Nigeria Enugu Campus, Enugu, Nigeria.; ^7^Department of Sociology and Anthropology, University of Nigeria, Nsukka, Nigeria.; ^8^Department of Economics, University of Nigeria, Nsukka, Nigeria.; ^9^Veritas Universit, Abuja, Nigeria.

**Keywords:** Health Sector Corruption, Nigeria, Nominal Group Technique, Priority Setting

## Abstract

**Background:** Corruption is widespread in Nigeria’s health sector but the reasons why it exists and persists are poorly understood and it is often seen as intractable. We describe a consensus building exercise in which we asked health workers and policy-makers to identify and prioritise feasible responses to corruption in the Nigerian health sector.

**Methods:** We employed three sequential activities. First, a narrative literature review identified which types of corruption are reported in the Nigerian health system. Second, we asked 21 frontline health workers to add to what was found in the review (based on their own experiences) and prioritise them, based on their significance and the feasibility of assessing them, by means of a consensus building exercise using a Nominal Group Technique (NGT). Third, we presented their assessments in a meeting of 25 policy-makers to offer their views on the practicality of implementing appropriate measures.

**Results:** Participants identified 49 corrupt practices from the literature review and their own experience as most important in the Nigerian health system. The NGT prioritised: absenteeism, procurement-related corruption, under-the-counter payments, health financing-related corruption, and employment-related corruption. This largely reflected findings from the literature review, except for the greater emphasis on employment-related corruption from the NGT. Absenteeism, Informal payments and employment-related corruption were seen as most feasible to tackle. Frontline workers and policy-makers agreed that tackling corrupt practices requires a range of approaches.

**Conclusion:** Corruption is recognized in Nigeria as widespread but often seems insurmountable. We show how a structured approach can achieve consensus among multiple stakeholders, a crucial first step in mobilizing action to address corruption.

## Background

Corruption has been defined as the abuse of entrusted power, such that a person, group, or organization acquires undue benefits. These may be financial, material, or non-material.^1^ Health systems are especially susceptible,^2,3^ often with life threatening consequences. Yet corruption in the health sector is often seen as intractable. A first step in addressing this issue is to identify the spectrum of corrupt activities, the actors involved, and those who have a stake in tackling it.^4^ We describe how we achieved consensus on the most important types of corruption in the Nigerian health sector and potential ways to tackle them.

Corruption in any health sector is propagated and sustained by a complex web of interacting factors.^5-8^ It thrives where frontline workers are poorly paid and lack resources to meet the needs of their patients, in settings characterized by weak governance structures and processes, lack of transparency, and ineffective accountability mechanisms.^5-8^ It is especially common among those involved in procurement of resources where oversight is weak.^9^ Many types of health sector corruption can become normalized, through custom and practice, even while those involved accept, even if not openly, that it is unjust and risks health. It is facilitated by the invisibility, other than to those directly involved, of many healthcare interactions, compounded by power and information asymmetry between providers and consumers of care.^10^

Nigeria ranks 148th out of 180 countries on the Transparency International 2018 Corruption Perceptions Index.^11^ Its health sector has been identified as one of its most corrupt sectors.^2^ This has been attributed to weak governance structures and accountability.^12^ Several studies have implicated corruption in adverse health outcomes,^13^ and it features frequently in studies of barriers to effective care.^1,7,8,12,14^ It is now attracting much discussion in the Nigerian health policy arena, with the media portraying it as a threat to Nigeria’s achievement of the health-related Sustainable Development Goals.^15,16^ If these concerns are to be turned into action, however, it is necessary to achieve a consensus on the most harmful types of corruption and what can be done about them.

Traditionally, scholars and activists have advocated measures to improve accountability and transparency throughout the health system. These can be vertical or horizontal. Vertical approaches involve promulgating rules and regulations to create accountability, provide checks and balances, and both sticks (dismissal, fines, etc) and carrots (incentives, perks or recognition for complying with regulations, etc). Horizontal approaches include collective agreements, codes of conduct, or informal contracts between health workers and managers forged by professional organisations, labour unions, community champions or grassroots movements. However, the evidence of effectiveness of these measures is weak.^1^ Measures such as barring gifts from the pharmaceutical industry to health workers, enhanced internal control procedures in community health centers, and regularization of co-payments combined with action against informal payments have had mixed results.^1,17,18^

Context is important. Despite its intuitive appeal, measures to increase transparency and accountability may bring few benefits where the ability of authorities to enforce rules is limited and key actors see few advantages when rules are enforced.^19^ A more pragmatic approach drawing on political economy and institutional economics,^19^ provides two insights for future directions in anti-corruption interventions. First, some forms of corruption are much more detrimental to the functioning of public services than others, so health gains from targeting them are likely to be very high. Second, some are deeply entrenched, serving the interests of powerful individuals and groups, requiring major political change that is difficult to achieve.

We report a study that sought to redress corruption by synthesizing the evidence on health sector corruption in Nigeria, capturing the types of corruption and their main manifestations, as well as the factors that drive them. We then sought consensus on priorities for action, considering the significance of each type of corruption and the feasibility of solutions.

## Methods

The most detrimental corrupt practices in the Nigerian health system were identified systematically. Types of corrupt practice were prioritized and, finally, feasible strategies to address them were generated and discussed.

### Stage 1: Literature Review

We conducted a systematic review of the literature on corruption in the health sector in Anglophone West Africa. In brief, we searched PubMed, Researchgate, Hinari, and Google Scholar. Studies were included initially if they were: (1) published between 2007 and 2017; (2) focused on corruption within Anglophone West African countries; and (3) written in English or with an available English translation. The review identified 61 papers describing a variety of corrupt practices, with absenteeism, diversion of patients to private facilities, procurement irregularities, informal payments, and theft of drugs and supplies among the most prominent. From it we extracted the 50 papers covering Nigeria and reviewed them in detail to ascertain: (*a* ) types of corrupt practices that had been reported specifically in the Nigerian health system and evidence of their impact on service users; (*b* ) incentives/disincentives (including policies and regulatory frameworks) for corrupt behaviour among frontline health workers and health facility managers; (*c* ) strategies that constrain corrupt practices by frontline healthcare providers and their managers; and (*d* ) roles of powerful organizations, lobbies, networks, associations, and influential individuals who may enable or obstruct the enforcement of existing legal and regulatory frameworks. This review was then used to inform the next step, consensus development.

### Stage 2: Nominal Group Technique Exercise

A Nominal Group Technique (NGT) was employed to build consensus among frontline health workers on corrupt practices that were most detrimental to the functioning of the health system. NGT is a group consensus-building method that aggregates the opinions of individuals with experience of, or important perspectives on, the phenomenon. A NGT provides an opportunity to canvass diverse views and, through a series of steps, develop consensus.^20^ Its structured process helps to reduce the influence of dominant speakers in group interactions, ensuring that individual voices do not skew the debate. Hence, all participants are given an opportunity to contribute equally.

Twenty-one frontline health workers (15 males and 6 females) from Enugu state and the Federal Capital Territory Abuja participated in the NGT exercise. They were purposively selected to represent different categories of health workers in the three tiers of healthcare (primary, secondary, and tertiary). They included: medical doctors, pharmacists, nurses, midwives, radiographers, laboratory scientists, and physiotherapists. Signed informed consent was obtained from each participant before the exercise. The discussions were audio recorded, backed up with hand-written notes. A team of 3 experts with experience in using NGT for policy design facilitated different segments of the discussion.

The NGT exercise was conducted in Abuja, Nigeria, in April 2018 (Box 1). Participants first heard presentations of findings from the literature review and a summary of current debates on corruption and anticorruption strategies in health sectors of low- and middle-income countries. They were then briefed on the purpose of the study and the NGT was explained. Participants were asked to individually generate a list of manifestations of corruption (hereafter corrupt practices) they have witnessed or know to be occurring in Nigeria’s health sector (‘silent generation’). Contributions were made in a round robin style, going around the table three times. During each round, each participant was asked to mention only one corrupt practice from his/her list, which was written on a flipchart, noting any repetition. Participants were encouraged to simply describe it and not to debate how prevalent or problematic it was at this stage. Each idea received a score of ‘1’ if different from previous suggestions. At the end of the third round, a list of 49 corrupt practices was derived, and participants were asked to clarify some of the concepts and wording rather than discuss their reasoning behind the ideas, thus reducing the chance of unconscious bias.

**Box 1. Nominal Group Process**

1. **Introductions and background** (10 minutes) – overall.
2. **Silent generation** – (15 minutes)- participants write their list of corrupt practices on postcards without interacting with other participants.
3. **Round Robin** – 1 idea per person up to 3 rounds (no more) (30 minutes) Facilitator write up suggested corruption types on a flip chart, research team members to write into computer.
4. **Clarification of ideas with participants** (20 minutes) – Facilitators condense ideas and transfer to a computer for slide presentation.
5. **Re-present the condensed options as a list** – condensed versions (5-10 minutes) up to 10.
6. Participants write down top 5 options silently on a card (5 minutes).
7. **Silent ranking** – done individually, each participant ranks for ‘most important and most feasible to address’ (10 minutes).
8. **Collect index cards**– partners to add data to the computer (10-minute break for participants).
9. Start of discussion while rankings are aggregated (20 to 45 minutes).
10. **Re-ranking results** – from original list (15 minutes).
11. **Present results** – display computer screen.
12. **Final discussion** – guided by facilitators.

The initial list of 49 corrupt practices was refined and condensed by merging and linking similar ideas. Participants were asked to reflect whether the condensed list represented the true picture of practices identified and to include any practice that was missed. Having agreed on a comprehensive list of 19 distinct corrupt practices, each participant was asked to select 5 and to rank them (on index cards) from most important to least important, considering their significance and harm (prevalence and impact on health). Votes were entered into an Excel spreadsheet and the top 5 types of corrupt practices were automatically generated using an algorithm. Numerical scores were generated by multiplying number of votes by weights based on reverse order of rankings. Thus, if ten participants ranked absenteeism first among the top five, the weighted score would be 10 × 5 = 50. If 8 participants ranked procurement-related corruption in fifth position, the weighted score would be 8 × 1 = 8. The totals for each type of corruption were then summed.

The aggregate initial rankings were presented to participants for discussion and clarification of inconsistent results. In the final stage, participants were asked to re-rank their original top-five ideas, this time, based on how easily they can be addressed (given existing political and institutional contexts). Their responses were again entered into an Excel spreadsheet and the summary scores computed. Changes in aggregate rankings were computed and fed back to participants.

### Stage 3: Stakeholder Workshop

We conducted a stakeholder workshop the following day with senior healthcare managers and policy-makers to validate the findings, to explore further the drivers of corruption in the health sector, and to operationalize measures to combat corruption among frontline health workers. Using a structured process of prioritizing with discussion, working within small groups, we focused on the manifestations of corruption. The workshop thus provided an opportunity for participants to reflect on: (1) the ranked list of corrupt practices among frontline health workers, (2) socioeconomic, political and institutional drivers of these corrupt practices, (3) anticorruption measures that have the potential to succeed, and how they could feasibly be implemented given existing policy and regulatory frameworks, and (4) powerful individuals or groups whose positionality could enable or obstruct enforcement of anticorruption measures.

Twenty-five participants (19 male and 6 female), comprising senior healthcare managers and policy-makers from government organizations/agencies, and representatives from international organizations and bilateral agencies, attended the workshop. These included the Federal Ministry of Health, Enugu state Ministry of Health, FCT Department of Health, National Primary Health Care Development Agency, National Health Insurance Scheme (NHIS), World Health Organization (WHO), and United States Agency for International Development, as well as representatives of 2 anti-graft agencies, the Independent Corrupt Practices Commission and Economic and Financial Crimes Commission.

The one-day workshop consisted of informative presentations, a group activity, and a plenary discussion. Having informed participants of the purpose of the study and objectives of the workshop (“to reflect on prevalent corrupt practices among frontline health workers in Nigeria” and “to identify practical and feasible interventions for curbing these practices”), the findings from the narrative literature review and NGT were presented. Participants were then assigned randomly to three groups (A, B, and C) for facilitated participatory discussion about drivers of specific corrupt practices and measures for mitigating or preventing their occurrence. The groups focused on the top 5 corrupt practices identified in the NGT process the previous day. Feedback from groups was followed by plenary discussion about the feasibility of implementation of suggested anti-corruption measures, and potential influence of powerful groups and individuals.

Findings from the workshop were synthesized and compared with findings from literature review. Consistencies and inconsistencies across methods are reported in subsequent sections of this paper.

## Results

### Literature Review

Most of the 50 studies concerning Nigeria were published after 2010. The largest share involved quantitative surveys while a few made use of qualitative data from in-depth interviews, observations, documentary reviews and other exploratory methods. For the present study we focused primarily on the qualitative studies identified in that review as they provided insights into the drivers of corruption most relevant to our objectives, harnessing rich information from lived experiences of providers, their managers, and clients. Table 1 summarizes characteristics of the top five recurring corruption concerns. Only a few studies actually evaluated the measures proposed.^17,21^ We summarise the measures identified in Box 2.

**Box 2. Suggested Approaches to Reducing Corruption reconsidered this (moving from fifth to third). Conversely,**

**a. Whistle blowing:** Tormusa and Idom recommended whistle blowing as a means to encourage reporting of misconduct, fraud and corruption, but it should be accompanied by effective protection for whistle-blowers.^8^
**b. Strengthening audit systems:** Vian recommended creation of fraud control units, training of internal auditors, and surveillance systems to tackle corruption, coupled with interventions to educate or change beliefs.^10^
**c. Sector-specific anti-corruption agency:** The establishment of an independent agency to investigate and enforce efforts against overbilling, bribes, etc was suggested.^1^
**d. Strengthening community governance systems:** Several authors identified lack of accountability as a factor encouraging corruption, leading to suggestions that citizens could act as watchdogs. Unlike many of the other suggestions, this was supported by evidence that regular community monitoring in public hospitals was effective in checking absenteeism.^17^ Mooketsane and Phirinyane pointed to weak governance and mismanagement of funds as key processes sustaining corruption in the health sector and so recommended involving churches in management of health facilities, arguing that their moral standards would reduce corruption.^6^ However, this is likely to depend strongly on context.
**e. Increase co-payments for free health services:** It was suggested that informal payments could be displaced by formalising co-payment for free health services.^17^
**f. Improved remuneration of health workers and adequate supply of work material/tools:** Several authors note the low pay of health workers and advocate monetary incentives dissuade them from taking bribes,^26,28^ although some note that this should be accompanied by measures to ensure adequate and dependable supplies of equipment and commodities (particularly drugs).
**g. Publication of performance data:** The need to monitor and supervise the activities of public health facilities to prevent diversion of funds, drugs and medical products cannot be overemphasized.^1^
**h. Strengthening procurement monitoring systems:** These include increased surveillance of stores, improved staffing (including security personnel) and weekly/monthly review of records and inventory, coupled with improved hiring processes and monitoring of staff. Suggestions to tackle corruption in tendering processes included, use of (electronic) media in publicizing and updating tenders and supplies, deployment of management information systems in monitoring the flow of supplies, and regular internal and external audits.
**i. Other recommendations:** Some authors highlighted the role of collective beliefs/norms/expectations regarding the giving and taking of bribes in Nigeria, and suggest that interventions that target addressing belief systems and norms are critical for countering health sector-related corruption.^5^ Others include awarding performance bonuses, legislation to make managers legally responsible for actions of subordinates, and regular monitoring to ascertain the official and actual fees paid by service users.

**Table 1:** Types of Corruption Reported in the Nigerian Health System

**Corruption Type**	**Manifestations**	**Drivers**
Bribery and informal payments	Bribes taken to let patients jump queues^22^; Give priority treatment to patients^23^; Charges for supposed free services^24^; Bribes taken to cover erring staff and fast-track promotion of health workers.^25^	Normalization of bribery by service users who gain quicker services^5^; Prevalence of out-of-pocket payments^26^; Poor pay of health workers^1^; Absence of an automated system of payment.^24^
Absenteeism	Health workers not turning up for work at all^13^; Turning up late^27^; Leaving workplace before closure time^28^; Deliberate idleness at workplace.^12^	Weak rules that check absenteeism^27^; Transport difficulties/geographical location of facility^29^; Poor pay of health workers to fund transport cost^24^; Political protection against sanctions^25^; Dual and private practice.^30^
Theft/diversion of money, drugs and medical supplies	Selling supplies for public consumption privately, and with extra cost^31^; Selling substandard products to patients while retaining quality ones for private sales^27^; Withholding free hospital supplies from patients and selling self-owned supplies in place of free supplies^31^; Embezzlement of healthcare funds.^5^	Dearth and weak enforcement of existing consumer protection laws^17^; Prevalence of out-of-pocket payments^26^; Ignorance of service users.^32^
Drugs and medical equipment procurement malpractices	Supply of substandard products by contractors^28^; Political considerations in securing procurement contracts^12^; Illegal sales of supplies to facilities for private profits^25^; Releasing seized substandard consumables after collection of bribery^33^; Taking kickbacks to prescribe and sell a particular product to patients, even if not appropriate^33^; Impersonation of licences by non-pharmacists.^33^	Absence of consumer protection measures^17^; Weak enforcement of procurement laws^33^; Poor understanding of procurement processes by staff and poor record keeping and store management^25^; Inappropriate cordial relationships between health agencies and hospital management boards^13^; Weak monitoring mechanisms.^33^
Diversion of public facility patients to private facilities and vice-versa	Refer patients from public facilities to private facilities where they gain from exorbitant charges^28^; Use equipment in public facilities to treat private patients.^34^	Health workers’ poor pay^29^; Political protection of doctors.^35^

### Consensus Development Exercise

The findings from the literature review were presented to participants as described above. Participants added several practices that had not featured prominently in the literature, including document forgery, falsifying information for private gain, favouritism/nepotism in employment, promotions and deployment, undertaking treatments beyond the expertise or authorisation of the practitioner, deliberate underpayment of medical staff, job purchasing and corruption in training, prioritizing activities that are beneficial for workers, failure of accountability for unfinished projects, and infiltration/trading of counterfeit drugs. This discussion generated a list of 49 corrupt practices (Table 2).

**Table 2:** Corrupt Practices Generated From the Round Robin Session

1	Unhealthy practices in employment of health workers	26	Inappropriate exemptions of health services
2	Unlawful receipt of money from patients	27	Connivance with patients for personal gain
3	Diversion of patients from public hospitals to private facilities	28	Undue reimbursement or claims
4	Impersonation of doctors by other health workers	29	Playing politics with patients
5	Inappropriate prescribing	30	Hoarding of bed-space for personal patients
6	Procurement of illegal drugs	31	Overpricing drugs
7	Procurement of equipment that are not needed	32	Invoicing fraud
8	Favouritism/Nepotism	33	Mal-distribution of health workers
9	Bringing private patients into public hospitals and charging them as private patients	34	Delay in reporting data as required
10	Negligence of patients	35	Increasing hospital bills without commensurate services
11	Diversion of medical supplies to private facilities	36	Stealing or exchanging babies for fee
12	Use of proxy patients	37	Giving and taking kickbacks
13	Requesting for payments for free services	38	Falsification of data
14	Protecting members of professional bodies even when they have committed crimes	39	Refusal to attend to patients based on financial constraints
15	Provision of fake documentation	40	Refusal to stepdown acquired knowledge
16	Over-budgeting	41	Not following procurement procedures to get supplies
17	Giving and taking kickbacks	42	Poor attitude of health workers
18	Lateness to work/absenteeism	43	Late arrival to work
19	Dual appointments	44	Deliberate late release of funds
20	Use of hospital vehicles for private businesses	45	Poor leadership
21	Falsification of results for private gain	46	Theft of consumables
22	Printing of fake receipts	47	Delay in payments of health workers
23	Procurement of fake drugs	48	Sell of substandard medicines
24	Ghost workers	49	Lack of funding and no proper accounting systems
25	Sales of personal consumables in public facilities		

The 49-item list was discussed among participants and facilitators and condensed into 19 corrupt/illicit practices by merging or removing duplicates and overlaps. Participants then ranked them in terms of which were most important, in terms of significance and harm, followed by feasibility of addressing them. The top five corrupt practices that emerged (with their weighted scores) were: absenteeism (53), procurement-related corruption (34), under-the-counter payments (33), health financing-related corruption (28), and employment-related corruption (26).

After reflection and discussion of the ranked items, participants re-ranked the items. Table 3 shows the results of each round of ranking. In the second ranking, absenteeism (82) ranked highest. This was followed by under-the-table/informal payments (71); employment-related corruption (59); health financing at facility level (54); and procurement-related corruption (42). There were some shifts between the ranking stages. Thus, while informal payments were considered less feasible to address in the first round of ranking, discussions highlighted strategies that may offer potential (with the rank moving from 3 to 2). Similarly, employment-related corruption was not initially seen as tractable given the need for a fundamental institutional change in regulations and enforcement mechanisms but, after debate, participants reconsidered this (moving from fifth to third). Conversely, procurement-related corruption was de-prioritised when the strength of opposition from vested interests (powerful groups and actors) was discussed.

**Table 3:** Results From NGT Voting Exercise With Frontline Health Workers

**Condensed List of 19 Corrupt Practices (2nd Listed)**	**Initial Ranking - Based on Significance and Harm (Weighted Scores)**	**Re-ranking – Based on Feasibility to Address (Weighted Scores)**
1. Employment-related corruption	1. Absenteeism (53)	1. Absenteeism (82)
2. Under the table payments	2. Procurement-related corruption (34)	2. Under-the-table/informal payments (71)
3. Diversion of patients from public hospitals to private facilities	3. Under-the-table/informal payments (33)	3. Employment-related corruption (59)
4. Procurement of illegal drugs	4. Health financing-related corruption (28)	4. Health financing corruption at facility level (54)
5. Procurement-related corruption	5. Employment-related corruption (26)	5. Procurement-related corruption (42)
6. Bringing private patients into public hospitals and charging them as private patients		
7. Negligence of patients		
8. Diversion of medical supplies to private facilities		
9. Use of proxy patients		
10. Requesting for payments for free services		
11. Protecting members of professional bodies even when they have committed crimes		
12. Over-budgeting		
13. Giving and taking kickbacks		
14. Absenteeism		
15. Dual appointments		
16. Provision of fake documentation		
17. Procurement of equipment that are not needed		
18. Health financing at facility level		
19. Lateness to work		

Abbreviation: NGT, Nominal Group Technique.

### Stakeholder Workshop

The senior managers and policy-makers concurred with all the findings from the NGT but highlighted their real-life drivers and significance, and suggested some actionable steps to implement them. Below are the outcome of the reflections from the workshops sorted in their different groups (A, B, C).

### Group A – Absenteeism and Under-the-Table Payment

Absenteeism was conceptualized broadly as being absent from the workplace, arriving late, and leaving before closing time. There was some debate about whether consciously not performing or refusing to perform tasks/duties when in the workplace was a form of absenteeism, with group members taking differing views. Participants agreed that it was widespread across the health sector but most prevalent within primary care and among more senior health workers – who were more often absent than the junior ones. Similarly, under-the-table-payments were considered a problem at all levels of healthcare but most prevalent in secondary and primary levels of care. The practices involved included extra-billing of clients or unauthorized (‘illegal’) payments made at a range of service points in a health facility and the ‘parallel sale’ of drugs and other consumables to clients.

Participants argued that weak governance, lack of supervision, poor attitude (often driven by the stresses of working in the system) and poor remuneration within the health system drove health workers to seek money in other ways, including absenteeism and under-the-table payments. Other drivers of absenteeism in primary care include underutilization of (low demand for) primary health centres for health services. However, health facility administrators and some other influential workers were considered to benefit from this type of corruption so it might be very difficult to facilitate change through measures that relied on their involvement. In these situations, stakeholders considered that top down or vertical interventions by the government, in the form of legislation and enforcement of rules would be necessary.

Absenteeism was also facilitated by structural problems, such as poor transportation and the long distances that health workers often have to travel to work. Female health workers often had competing family responsibilities. Senior doctors (consultants in particular), regardless of gender, had high social status that led to expectations that they would provide financial support to relatives beyond what was possible on their official income, leading them to seek additional income streams (dual practice) that required absence from facilities.

Stakeholders identified some technical remedies such as; clock-in/clock-out systems, linked to sanctions and rewards. Rewards might include cash incentives, display of pictures of especially committed staff, and greater flexibility in permitting authorised absences when needed. Structural interventions included improving the provision of accommodation and transportation for health workers and providing better equipment in health facilities, which would have direct benefits for patients beyond any impact on staff satisfaction. Views on public-private partnerships, such as those that would permit health workers to operate in both sectors in the same facilities, differed. Some saw this as a way to retain them in the facilities while others were concerned that it would create complex arrangements that would be prone to exploitation and abuse, thereby increasing corruption. As the participants discussed the feasibility of introducing these interventions, many, questions were raised but not answered about what changes would be needed in funding, policies, and legislation. Participants found it difficult to see how they would be able to convince authorities to make any changes, given the potential opposition they would face for uncertain personal or political benefit.

Discussions about under-the-table payments included several bottom up approaches. Increasing patient involvement could, it was thought, empower them by virtue of improved knowledge and strengthening the voice of service users, especially in relation to official prices of medical consumables and services. Stakeholders also proposed increasing choice, allowing patients to switch consultants and healthcare teams when irregularities occur. It was also suggested that costing and dispensing of consumables be decentralised, with trusted and reliable reporting platforms being established.

Reducing under-the-table-payments was also identified as an important step in increasing revenue in health facilities, creating an incentive for health centre management teams to stamp this out. Again, however, the group thought that top down mechanisms would be necessary and would include audits, new legislation to support transparency in costing and dispensing consumables, protection for whistleblowing, and stiff sanctions for defaulters. Given the financial benefit that these could create for the health system, the participants considered that these would be more likely to gain buy-in from senior members of the government (in comparison to the suggestions around absenteeism).

### Group B – Procurement-Related Corrupt Practices

The second group discussed procurement-related corruption, much of which related to the distribution and theft of pharmaceuticals. A range of actors was identified as involved, including sales representatives, doctors, auditors and pharmacists. It was believed that these activities required a network of complicit actors.

Participants contended that curbing this form of corruption required that the procurement system be fully digitalized with effective recording, monitoring and feedback systems. They also suggested that improving clients’ awareness of essential medicines list and prices, as well as institutionalizing sanctions against offenders. Other potential anticorruption measures include: promoting a drug-revolving fund system, improving the tendering process by having multiple simultaneous submission and using limited access tendering boxes. The introduction of Information and communications technology for proper data management and monitoring the flow of supplies was also considered necessary to check for procurement corruption. Measures that would encourage institutions to adhere to procurement rules and whistleblowing policies were also identified as important. However, participants all agreed that most measures would require new legislation, imposing a vertical approach. Some horizontal measures considered feasible include measures to help patients – understand pricing systems and creation of facility based committees to oversee procurement, usage, and supply of medical consumables. Such committees should have representation by patients and involve staff, especially those with high moral standards and self-discipline. Nevertheless, the suggested horizontal measures all would require enabling vertical ones, especially legislation, to operate. This was considered challenging.

### Group C – Corrupt Practices Related to Employment and Health Financing

The third group discussed employment- and health financing-related corrupt practices. Examples include: malpractice perpetuated by health maintenance organizations (HMOs) and service providers within the NHIS such as irregular reimbursement of fee-for-service by HMOs; billing HMOs/NHIS for services not provided to clients; extra-billing of insured clients; lack of update of enrolee lists by HMOs; and hoarding of drugs and false reporting of stock-outs of NHIS drugs in health facilities. Others mentioned included issuing fake receipts to clients and failure to release budgeted funds to health facilities. These practices, considered as involving very powerful groups and persons, were also said to be driven mainly by weak governance and regulatory oversight of NHIS, poor/unpredictable budgets for the health sector, and poor planning and prioritization.

Suggested strategies for curbing employment-related corruption mostly involved regulatory measures enacted and enforced by high-level authorities. Thus, vacancies should be advertised and the best applicants selected, rather than as is often the case using patronage; conducting staff audits using benchmarks for competence; deploying health workers appropriately; and ensuring a sustainable supply, with younger health workers being mentored and rising through ranks on the basis of their expertise. These measures were seen as creating a merit-based system that outlives the immediate future. Such an approach should curb irregularities as incompetent workers would become frustrated with the system, creating a deterrent for unqualified persons yet to be employed.

The only horizontal measures, involving different institutions and actors, and civil society groups, were empowerment of communities to demand deployment of health workers that have the skills required to meet their health needs. Proposals to tackle corruption in health financing were dominated by suggestions to improve efficiency of the NHIS through better regulation of HMOs, accreditation and improved oversight of service providers, annual updates of enrolee lists, and strengthening accountability of service providers to enrolees. Suggested approaches include detailing of services provided to clients on an invoice which must be signed by the client before forwarding to HMOs for reimbursement; establishment of effective channels for client complaints and feedback to NHIS; and public display of pricing list of services.

In summary, excluding employment-related corruption, the most promising anti-corruption strategies are those that could be implemented using both top down and horizontal approaches (at the grassroots level) and involve collective agreements and actions by key actors influential at the service delivery level. While there was perception that initiatives by grassroots organisations or movements can play a major role, if they are to rise to the challenge of tackling corruption they will have to be strengthened. It is thus important for civil society organisations, non-governmental organisations, and other bodies with a stake in combatting corruption to encourage and lead grassroots efforts to undertake these strategies.

##  Summary, Limitations, and Outlook

Corruption in the Nigerian health system is commonplace and a serious issue but, too often, is placed in the “too difficult” tray and the evidence on its scale and nature and on responses that have been adopted is often fragmentary. We have shown that it is possible to reach agreement among frontline health workers and policy-makers about specific types of corruption that are significantly harmful yet could be feasibly engaged. Our approach was participative, listening to those who have first-hand experience of this phenomenon. With them, we were able to develop a list of five priorities for action. These are: (1) absenteeism; (2) under-the-table/informal payments; (3) employment-related corruption; (4) health financing corruption; and (5) procurement-related corruption. These were broadly consistent with what we had found in the literature.^8,12,26,31,34^ Thus, there are accounts of procurement-related corruption and health financing corruption driven by political factors with involvement of senior and top health managers in Nigeria.^12,31^ Absenteeism by health workers has also been reported in several parts of the country, with sickness and poor social and physical environment identified as underlying factors.^36,37^ There is also evidence of informal payments in Nigeria, specifically in relation to malaria treatment.^26^

However, we went beyond simply creating a ranked list of problems. Policy-makers who are aware of the political and economic realities of the Nigerian context, appraised each type of corruption to generate potential feasible measures that can be implemented to reduce or stop the listed types of corruption.

One surprising lesson can be learnt from our approach and findings. Corruption in Nigeria is often conceived to be grand embezzlement and/or heavy pilfering of public funds by high ranking government officials. Yet our findings reveal widespread *everyday* corruption involving frontline health workers (eg, doctors, nurses, pharmacists, accountants, etc), whose actions/inactions have direct and serious consequences for the delivery of healthcare. Some earlier studies tend to discuss these types of corruption as challenges facing the health sector.^26,36^ Our approach to identifying and discussing the types of corruption explicitly consider the practices as corruption and helped raised consciousness that perpetrators are often frontline staff.

We do not deny that large scale embezzlement happens, or that it has significant impacts on health outcomes, but frontline health workers as well as policy-makers realize that such types of corruption are possibly beyond their influence. Crucially, health workers believe that there are feasible solutions that can be implemented at the grassroot level. Such measures avoid the legislative inertia that would make many top-down anti-corruption strategies challenging.

Our study has several limitations. First, it was not feasible to include health workers from all regions of Nigeria. With only 50 participants (including health workers and policy-makers), we may not have captured the entire spectrum of corruption prevalent in Nigeria and we may have given undue prominence to corrupt practices dominant in the places from which participants were drawn. Future studies could replicate this study in other regions of the country to see if similar or more diverse forms of corruption will be identified. Our study was exploratory and was unable to examine in-depth, the specific forms of corruption identified. Further research using qualitative methods such as in-depth interviews and focus group discussions would help to understand in more depth the drivers of various forms of corruption and their dynamics.

However, this is the first study in the region to employ structured consensus building to prioritise action on forms of corruption, taking account of what is feasible. It was not obvious that this would have been possible at the outset given the sensitivities involved. In this way it offers a means by which researchers and policy-makers in other low and middle income countries can begin the process of tackling corruption. It is important to stress the contribution of engaging in a process of consensus building. It would have been possible to derive the final list of priorities simply from the literature. However, by engaging in discussion, participants changed their views about what was possible, identifying and supporting potential measures they would once have discounted. The approach we took also enabled those at the frontline and those at the apex of the system to engage with one another in a constructive manner that otherwise does not occur.

Our experience provides support for targeted, pragmatic approaches to governance and anti-corruption in health systems policy. Such approaches are gaining ground in other sectors (education and industrial policy) where, under the rubric of developmental governance,^38^ or cumulative incrementalism,^39^ actors are seeking ways to intervene “within the grain,”^40^ that is by taking account of the ways in which the economic, political and social structures limit the potential for action. Such approaches recognise that the long-standing search for comprehensive solutions to corruption in society, or more narrowly in the health sector, has not, in general, proven fruitful. In health, the enormity of the task, the multiplicity of actors involved, and the limited institutional capacity for reform have led to corruption being seen as impossible to tackle at the current stage of development. Politicians in many countries have entered government with a stated intention to do something about it but failed. This was borne out in our discussions with key stakeholders. There was widespread agreement about the need for stronger systems of governance, accountability, and transparency, but the practical steps that needed to be taken remained elusive. In contrast, by focusing on specific manifestations of corruption, those who participated in this exercise were able to identify a number of measures that did seem feasible and offered potential for success. In a number of cases, individuals who began the process with a degree of scepticism about what could be achieved changed their minds.

Obviously, the ideas generated now need to be further operationalised in policies and interventions that must be evaluated, not least because of the risk of unintended consequences. Thus, while some measures (eg, flexible working conditions and improved accommodation/transport for health workers) seem relatively straightforward, even if requiring additional resources, others (eg, the introduction of sanctions and rewards for dealing with absenteeism) will need to be carefully designed and evaluated to ensure that they are achieving their intended goals.

Some of the measures proposed lie within the scope of district/local authorities, who have the power to implement them. Some other measures require relatively simple changes, such as improved documentation of financial flows. However, others require action at a higher level. Participants supported managers and policy-makers working concert to promote appropriate legislation.^41^ Jackson and Köbis suggest that a multipronged approach that will counter both vertical and horizontal pressures to be corrupt and mobilize local action will be more effective in fighting corruption within communities and organisations.^42^ For instance, effective feedback mechanisms (such as phone-in centres or hotlines) for patients to report informal/under-the-table payments would require supportive ‘formal’ health financing or consumer protection policies. Similarly, whistleblowing would only produce results if policy and political environments enable enforcement of sanctions.

The identification of practical measures that can be taken should not, however, divert attention from the larger issues underpinning the persistence of corruption, including inadequate resources for the health sector.^12,30,43,44^ Inadequate government funding leads to low wages for health workers, poor infrastructure and basic amenities, and lack of equipment and consumables in health facilities, all creating the conditions for corrupt practices. Onwujekwe et al found that health workers who demanded informal payments from clients for treatment of malaria, which was supposed to be free, did so to augment their salaries and generate internal revenue to keep the facility running.^26^ In addition to low wages, health workers in the public sector lack materials (including medicines) and equipment to deliver quality healthcare.^44,34^ Some health workers are expected to work in health facilities for several years as unpaid volunteers.^45^ Addressing these issues will require structural changes and better resourcing of the health system which need to be addressed over a longer time period.

Despite the lack of evidence on the effect of transparency and accountability initiatives on corruption, there is some evidence that horizontal approaches involving communities monitoring and participating in decision-making has led to improvements in local governance and health outcomes.^46^ There is a clear need to empower patients. One explanation for the persistence of informal payments, developed by Gaal and McKee,^47^ is that they represent a means of informal exit (INXIT), in settings where patients lack both exit (to alternative services) and voice (redress). Hoffman and Patel show how a failure by patients to question medical staff who accept corrupt practices encourages corruption in the health sector.^5^ However, this will require considerable efforts to strengthen civil society groups and create a political space where the voice of multiple actors can be heard.

In summary, although only a first step, this exercise presented in the paper has shown that it is possible to bring together people from across the health sector in Nigeria to discuss corruption, and to conclude that some solutions are possible, even if it will take some time to fix the underlying problems. We hope to take this research further, building on a more detailed understanding of the drivers of the corrupt practices identified, while exploring the feasibility and effectiveness of the solutions suggested. We hope that those studying corruption in other countries will find our approach useful and utilize it to identify and engage the prevalent damaging types of corruption in their contexts. Countries in the same region with Nigeria, especially Anglophone West African countries, may build on the findings from our systematic reviews (see full details in Onwujekwe et al),^48^ to begin to explore and prioritise the top health sector corruption prominent in literature in their countries.

## Acknowledgements

This paper is from a larger multi-country anti-corruption evidence study in Bangladesh, Nigeria and Tanzania by the School of Oriental and African Studies at the University of London, UK, in partnership with the London School of Hygiene and Tropical Medicine, UK, and the University of Nigeria, Nsukka (Enugu-campus). The anti-corruption evidence research consortium is funded by the UK Department for International Development. We are grateful to the participants in the NGT for their involvement in the study.

## Ethical issues

Ethical approval was granted by the Health Research Ethics Committee of the University of Nigeria Teaching Hospital, Ituku-Ozalla, Nigeria (Approval No: NHREC/05/01/2008B-FWA00002458-IRB00002323).

## Competing interests

Authors declare that they have no competing interests.

## Authors’ contributions

Conception and design: OO, EH, DB; Acquisition of data: OO, CTO, PA, AD, PO, CM, EH, HI, UO; Analysis and interpretation of data: Cm, OO, AO, PA, CTO, UO, PO; Drafting of the manuscript: OO, CTO, MM, PA, DB; Critical revision of the manuscript for important intellectual content: MM, DB, EH, OO; Obtaining funding: OO, DB, EH, MM; Supervision: OO, DB, MM.

## Authors’ affiliations

^1^Health Policy Research Group, College of Medicine, University of Nigeria Enugu Campus, Enugu, Nigeria. ^2^Department of Health Administration and Management, College of Medicine, University of Nigeria Enugu Campus, Enugu, Nigeria. ^3^Department of Psychology, University of Nigeria, Nsukka, Nigeria. ^4^London School of Hygiene and Tropical Medicine, London, UK. ^5^Department of Social Work, University of Nigeria, Nsukka, Nigeria. ^6^Department of Community Medicine, College of Medicine, University of Nigeria Enugu Campus, Enugu, Nigeria. ^7^Department of Sociology and Anthropology, University of Nigeria, Nsukka, Nigeria. ^8^Department of Economics, University of Nigeria, Nsukka, Nigeria. ^9^Veritas Universit, Abuja, Nigeria.

## Key messages

**Implications for policy makers**

- Though corruption may seem intractable, identifying the most damaging types of corruption and their drivers is a crucial step in tackling corruption.
- Our Nominal Group Technique (NGT) approach blended contributions from frontline health workers, top health managers, policy-makers, and diverse stakeholders including anti-corruption and international agencies, to gain a panoramic understanding and proffer solutions to health-sector corruption in Nigeria.
- Findings support that the application of vertical and horizontal approaches is necessary to reduce corruption in the health sector.

**Implications for the public**

Corruption in the health sector contributes to poor service delivery which amounts to the loss of lives and other resources. Developing countries such as Nigeria often face the challenge of corruption and the hopes of eliminating corruption are often low. Our study involved health workers who identified the specific types of corruption that are very damaging to the health sector in Nigeria. Health workers also indicated the types of corruption that can be easily tackled, and ranked them in the order of priority. We got policy-makers to discuss these forms of corruption, and offer potential solutions that could tackle them. Solutions to the identified corruption types involved actions that can be driven by government structures/ authorities and actions that can be enforced by horizontally by everyday people.
